# Resource utilization in patients with schizophrenia who initiated risperidone long-acting therapy: results from the Schizophrenia Outcomes Utilization Relapse and Clinical Evaluation (SOURCE)

**DOI:** 10.1186/1471-244X-11-168

**Published:** 2011-10-14

**Authors:** Concetta Crivera, Cherilyn DeSouza, Chris M Kozma, Riad D Dirani, Lian Mao, Wayne Macfadden

**Affiliations:** 1 Janssen Scientific Affairs, LLC, Raritan, New Jersey, USA; 2 Veterans Affairs Medical Center, Kansas City, Missouri, USA; 3 University of South Carolina, Columbia, South Carolina, USA; 4 Janssen Scientific Affairs, LLC, Titusville, New Jersey, USA; 5 Johnson & Johnson Pharmaceutical Research and Development, LLC, Titusville, New Jersey, USA

## Abstract

**Background:**

Schizophrenia is a chronic mental health disorder associated with increased hospital admissions and excessive utilization of outpatient services and long-term care. This analysis examined health care resource utilization from a 24-month observational study of patients with schizophrenia initiated on risperidone long-acting therapy (RLAT).

**Methods:**

Schizophrenia Outcomes Utilization Relapse and Clinical Evaluation (SOURCE) was a 24-month observational study designed to examine real-world treatment outcomes by prospectively following patients with schizophrenia initiated on RLAT. At baseline visit, prior hospitalization and ER visit dates were obtained for the previous 12 months and subsequent hospitalization visit dates were obtained at 3-month visits, if available. The health care resource utilization outcomes measures observed in this analysis were hospitalizations for any reason, psychiatric-related hospitalizations, and emergency room (ER) visits. Incidence density analysis was used to assess pre-event and postevent rates per person-year (PY).

**Results:**

The primary medical resource utilization analysis included 435 patients who had a baseline visit, ≥1 postbaseline visits after RLAT initiation, and valid hospitalization dates. The number of hospitalizations and ER visits per PY declined significantly (*p *< .0001) after initiation with RLAT. A 41% decrease (difference of -0.29 hospitalizations per PY [95% CI: -0.39 to -0.18] from baseline) in hospitalizations for any reason, a 56% decrease (a difference of -0.35 hospitalizations per PY [95% CI: -0.44 to -0.26] from baseline) in psychiatric-related hospitalizations, and a 40% decrease (-0.26 hospitalizations per PY [95% CI: -0.44 to -0.10] from baseline) in ER visits were observed after the baseline period. The percentage of psychiatric-related hospitalizations decreased significantly after RLAT initiation, and patients had fewer inpatient hospitalizations and ER visits (all *p *< .0001).

**Conclusion:**

The results suggest that treatment with RLAT may result in decreased hospitalizations for patients with schizophrenia.

**Trial Registration:**

ClinicalTrials.gov: NCT00246194

## Background

Schizophrenia is a chronic mental health disorder associated with increased hospital admissions and excess utilization of outpatient services and long-term care [1]. In 2002, direct and indirect costs attributed to schizophrenia in the United States were $63 billion [1]. Although the lifetime prevalence of schizophrenia is approximately 1.5%, the cost of treatment accounts for nearly 2.5% of total health care expenditures in the United States [2]. Therapeutic intervention for schizophrenia includes use of antipsychotic medications in the acute phase of the disease, followed by long-term maintenance therapy. Many patients respond to initial treatment of first-episode schizophrenia but then experience a relapse, defined as rehospitalization, symptom re-emergence, or both. Relapse has been strongly associated with partial adherence or nonadherence to treatment [3]. Poor adherence to antipsychotic medications has also been associated with rehospitalization and higher hospitalization costs [4]. Therefore, to reduce health care costs incurred by patients with schizophrenia, adherence to therapeutic regimens should be improved. Treatments that reduce health care resource utilization in patients with schizophrenia have the potential to reduce overall health care costs substantially [4]. Such treatments may also reduce societal or indirect costs by reducing the care time required by caregivers and by providing patients opportunities to return to employment.

Risperidone long-acting therapy (RLAT), a second-generation injectable atypical antipsychotic approved for the treatment of schizophrenia, has been found to improve clinical symptoms and decrease relapse rates [5-9]. The injectable formulation of RLAT may facilitate improved treatment adherence, which can lead to improved patient outcomes and lower utilization of health care services. As conventional long-acting injectable antipsychotics have been found to decrease the one-year risk of hospitalization (21% to 36%) [10,11], previous studies suggest that RLAT may result in decreased hospitalization. In a 12-month study [7], 18% of patients were rehospitalized during the trial, while in another study, where 106 patients served as their own controls, hospitalization decreased to 42% (*p *< .001) after RLAT initiation compared with before initiation (75%) [12].

The Schizophrenia Outcomes Utilization Relapse and Clinical Evaluation (SOURCE) was an observational study designed to examine real-world treatment outcomes by prospectively following patients with schizophrenia initiated on RLAT. The health care resource utilization outcomes measures (per person-year [PY]) observed in this study were hospitalizations for any reason (defined as all-cause hospitalizations), psychiatric-related hospitalizations, and emergency room (ER) visits. The follow-up period for the SOURCE study was up to 24 months, in order to investigate clinical outcomes related to maintenance treatment with antipsychotic medications in patients with schizophrenia.

## Methods

### Study Design

The SOURCE project was a 24-month, multicenter, prospective, longitudinal, observational study conducted from September 2004 to January 2006 at 67 community mental health centers and Veterans Administration hospitals in the United States. The study protocol and subject informed consent form were reviewed and approved by an institutional review board. Patients eligible for enrollment were aged 18 years or older, were appropriate for initiation of RLAT, had a physician-based diagnosis of schizophrenia according to the *Diagnostic and Statistical Manual of Mental Disorders*, Fourth Edition *(DSM-IV)*, and had signed the informed consent form. Patients who were at imminent risk of injuring themselves or others or of causing significant damage to property, who were hypersensitive to RLAT or any of its components, or who had been treated with investigational agents within the previous 30 days were not eligible for enrollment.

The recommended initial RLAT dosage is 25 mg every 2 weeks by deep intramuscular gluteal injection. Patients not responding to 25 mg may benefit from a higher dose of 37.5 mg or 50 mg [13]. Patients were defined as having received RLAT if they had >1 record for RLAT in the injection log within 28 days before each study visit. This study had a naturalistic design: after enrollment, specific treatments or medical interventions were not mandated, so treatments for schizophrenia could have been stopped, started, or changed throughout the study, as deemed appropriate by the treating physician. Study site monitoring was not conducted. The safety and efficacy data from this study population has been described [14].

At the baseline visit, dates of prior hospitalizations and ER visits were obtained for the previous 12 months. At each 3-month visit, subsequent hospitalizations and ER visits since last study visit were obtained, if available, through patient or clinician reports. Hospitalizations were categorized as psychiatric-related (nonsocial reason) or "other"; ER visits were categorized as psychiatric-related or "other medical problem." Hospitalization for any reason (all-cause hospitalizations) were also determined and categorized as due to psychotic disease, social reasons, or "other." Dates of psychiatric events, defined as deliberate self-injury, clinically significant suicidal or homicidal ideation, or violent behavior resulting in clinically significant injury to another person or property damage, were also collected every 3 months.

### Statistical Methods

Each patient served as his or her own control. Patients were included in the analysis if they had a baseline visit, >1 postbaseline visit, and valid hospitalization dates. Because this was an analysis of a closed registry, no sample size calculation was performed for this analysis. All eligible patients were used in the analysis. The numbers of outcomes per PY prior to baseline (visit 1) and during all available postbaseline visits were calculated. When comparing resource utilization between the 12-month period before study entry (preperiod) and the postbaseline period, it was necessary to account for different lengths of follow-up times among patients. The incidence densities for hospitalization and ER visit were calculated. Incidence density was defined as the total number of events for the study population divided by the total length of time the population was at risk for these events in follow-up years. The bootstrap resampling method was used to calculate confidence intervals (CIs), and *p-*values were used to examine the difference between the prebaseline and postbaseline periods for hospitalization and ER visits.

A subgroup analysis was performed on patients with >2 RLAT injections who had valid dates in the prebaseline period. The percentage of patients with >1 hospitalization was evaluated for the prebaseline and postbaseline periods. Percentages were tested with a McNemar test.

Categorical measures were summarized using frequencies and percentages. Continuous measures were summarized using mean, standard deviation (SD), minimum, maximum, and median. SAS software (Version 9.1, SAS Institute Inc., Cary, NC, USA) was used for all analyses. All tests were two-tailed and conducted at the 5% significance level.

## Results

### Demographic Characteristics

A total of 532 patients were enrolled in the study at 66 study sites. Approximately 18% of patients did not return for the 3-month visit, and 13% did not return for the 6-month visit. At the 24-month visit, 210 (39.5%) patients remained in the study. A total of 435 patients who had a baseline visit, >1 postbaseline visit, and valid hospitalization dates were studied for the primary medical resource utilization analysis.

As shown in Table 1, the mean (SD) age of the study population (*n *= 435) was 41.9 (12.6) years, and 66.7% of the patients were male. The mean (SD) duration of illness was 17.6 (12.1) years. Twenty-two patients (5.1%) experienced an inpatient hospitalization at baseline. In this sample, 321 patients (73.8%) were initiated on the 25-mg dose of RLAT, 62 (14.3%) on the 37.5-mg dose, and 51 (11.5%) on the 50-mg dose (one patient had missing data). Additionally, in 38.9% of patients the investigator reported that they received other antipsychotics in addition to RLAT during the study. Of the patients in this sample, 419 (96.3%) had evidence of RLAT use at visit 1; of the 210 patients who attended visit 9, 160 (76.3%) had evidence that they were receiving RLAT.

**Table 1:** Demographic and patient characteristics

	N = 435
Age, y, mean (SD)	41.9 (12.6)^a^

Gender, male, %	66.7

Years since diagnosis, mean (SD)	17.6 (12.1)^b^

Hospitalized in prior year, %	39.3

Inpatient hospitalization at baseline, n (%)	22 (5.1%)

CGI-S score, mean (SD)	3.9 (1.2)

GAF score, mean (SD)	53.1 (13.7)^c^

CGI-S, Clinical Global Impression-Severity; GAF, Global Assessment Functioning; SD, standard deviation.

^a^*n *= 433; ^b^*n *= 427; ^c^*n *= 434.

For the subgroup analysis of the 343 patients who had received >2 RLAT injections and had a fixed observation period of 12 months, the mean (SD) age of the study population was 42.1 (12.8) years, and 66.5% of the patients were male. The mean (SD) duration of illness was 24.0 (8.6) years.

### Resource Utilization

**Figure 1:**
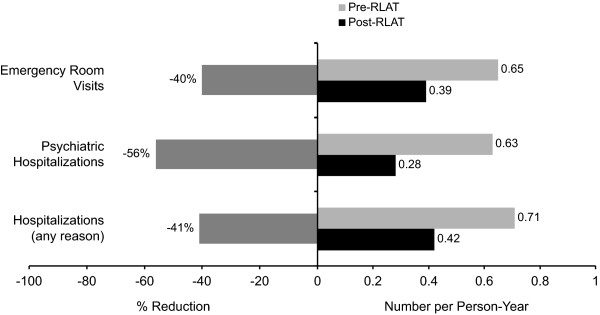
**Change from Baseline in Hospitalization Incidence Rate**. (pre-RLAT versus post-RLAT period [*n *= 435; *p *< .01 for all]).

**Figure 2:**
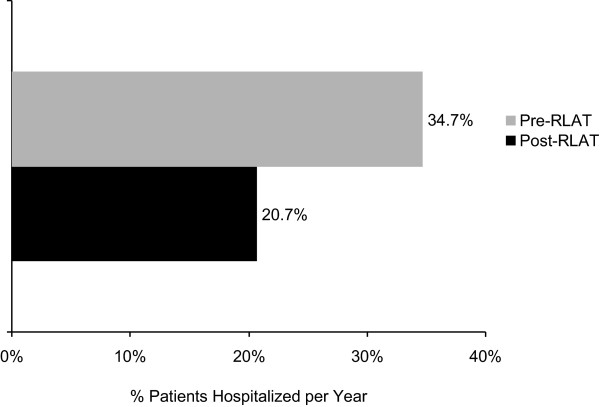
**Percentage of patients with >1 psychiatric-related hospitalization**. (*n *= 343; *p *< .0001).

The number of hospitalizations (all-cause and psychiatric-related) and ER visits per PY declined significantly (*p *< .0001) after initiation with RLAT. A 41% decrease (difference of -0.29 hospitalizations per PY [95% CI: -0.39 to -0.18] from baseline) in all-cause hospitalizations a 56% decrease (a difference of -0.35 hospitalizations per PY [95% CI: -0.44 to -0.26] from baseline) in psychiatric-related hospitalizations, and a 40% decrease (-0.26 hospitalizations per PY [95% CI: -0.44 to -0.10] from baseline) in ER visits were also observed after RLAT was initiated (Figure 1). Of those 343 patients who had received >2 RLAT injections and who had a fixed observation period of 12 months, the percentage of patients with >1 psychiatric-related hospitalization declined from 34.7% in the pre-RLAT period to 20.7% in the post-RLAT period (*p *< .0001; McNemar test; Figure 2).

## Discussion

Hospitalization is the major cost driver in the treatment of schizophrenia; 79% of the direct costs of schizophrenia are due to hospitalization or other residential care [4,5]. Results from SOURCE, an observational study that collected efficacy, resource utilization, and safety data of United States patients with schizophrenia for 24 months, demonstrated that the initiation of RLAT significantly reduced the number of hospitalizations for any reason and ER visits and decreased the percentage of psychiatric-related hospitalization by 14%. These results are in agreement with those of previous studies, which have shown that RLAT reduces hospitalization, ER visits, resource utilization, and inpatient bed days.

In the electronic Schizophrenia Treatment Adherence Registry (eSTAR) study for Spain [15], a higher percentage of patients (89.1% versus 67%) did not require hospitalization at 12 months after switching to RLAT, compared retrospectively with the patients in same period receiving the previous antipsychotic treatment. Similar results were observed at 24 months (85.2% versus 60%) [15]. In a retrospective study conducted in the United Kingdom, data on the number of hospitalizations and inpatient bed days were collected from 100 patients with schizophrenia, 12 months before and 12 months after initiation of RLAT [16]. A statistically significant reduction (*p *< .01) was observed in the number of hospitalizations in the 12 months after initiation of RLAT compared with the 12 months before initiation: 62 versus 22 admissions, respectively.

In a European study of 397 patients treated with RLAT [17], the number of patients requiring hospitalization decreased significantly (*p *< .0001) from 38% during the 12 weeks before study entry to 12% during the last 12 weeks of the study. The need for outpatient consultations also decreased significantly (*p *< .0001) from 70% during the 12 weeks before study entry to 30% during the first 12 weeks of treatment. The need for outpatient consultations remained stable during the remainder of the treatment period. No change in the number of ER admissions was observed before or during RLAT treatment.

Conversely, in a study in the United Kingdom, Young and Taylor found that switching to RLAT was associated with a continuing trend toward increased use of health care resources [18]. In this study, resource utilization data were collected for 3 years before and 1 year after RLAT initiation from 250 patients with schizophrenia. The mean number of days spent in the hospital per patient increased from 31 in year -3 to 44 in year -2, to 90 in year -1, and to 141 in year +1. The authors acknowledge that these results may reflect a selection bias because the patients studied tended to be more severely ill; the majority (69.6%) of these individuals were inpatients when RLAT was initiated. In fact, some studies suggest that RLAT can decrease hospitalization days. In a retrospective study at four sites in Germany using a mirror-image design [19], hospitalization rates and duration of inpatient treatment were assessed in patients with schizophrenia and schizoaffective disorder who were switched to RLAT treatment for 12 and 18 months, respectively. Patients who received RLAT had a mean of 0.53 and 0.49 inpatient hospitalizations at 12 and 18 months, respectively, compared with 1.51 hospitalizations prior to receiving RLAT (*p *< .0001). Patients who switched to RLAT also spent 27.4 and 38.4 fewer days in an inpatient setting at 12 and 18 months, respectively. Another, retrospective mirror-image analysis of all patients prescribed RLAT over a 35-month period in a United Kingdom psychiatric clinic also examined hospitalizations [20]. In this study, RLAT use compared with previous treatment was associated with a reduction in the number of hospital admissions (33 vs 65; *p *< .005) and total inpatient days (2188 vs 4550 days; *p *< .005). Mean RLAT treatment period in this study was 13.2 months.

As this was a nonrandomized, longitudinal, naturalistic, observational study, with no concurrent comparator group, there are several limitations that may influence the generalizability of the results. Patients were permitted to receive additional medications at the discretion of their clinicians and may or may not have received RLAT at the time of the study visit. Therefore, the reductions in health care resource utilization observed cannot be attributed to any particular treatment with certainty. However, although patients were permitted to receive additional medications, 76% who attended visit 9 were documented as having used RLAT, suggesting that RLAT might have contributed to the reduction in resource utilization. In addition, more than half the patients discontinued the study, suggesting potential selection bias for retaining patients to be responders to RLAT treatment. Additionally, data on hospitalization were obtained through patient and clinician reports; therefore, these data rely on the accuracy in reporting hospitalizations.

## Conclusions

In SOURCE, the percentage of patients hospitalized decreased significantly after RLAT initiation, and patients had fewer inpatient hospitalization and ER visits. Interestingly, the results of this study are similar to the results of several other studies that had a different study design or were conducted outside of the United States. Because of the high cost associated with hospitalization of patients with schizophrenia, these results suggest that the initiation of RLAT treatment could result in decreased health care costs.

## Competing interests

The authors of this manuscript: Concetta Crivera and Riad D. Dirani are employees of Janssen Scientific Affairs, LLC, and Johnson & Johnson stockholders. Cherilyn DeSouza declares that she has no competing interests. Chris M. Kozma is a consultant for Janssen Scientific Affairs, LLC. Lian Mao is an employee of Johnson & Johnson Pharmaceutical Research and Development, LLC, and a Johnson & Johnson stockholder. Wayne Macfadden was an employee of Janssen Scientific Affairs, LLC, at the time of this analysis.

## Authors' contributions

CD, CC, CMK, RD, and LM contributed to the conception and design, acquisition of data, analysis and interpretation of data, and drafting of the manuscript and its critical revision for important intellectual content. WM was involved in the interpretation of data, and critical drafting and revising of the manuscript for important intellectual content. All authors (CC, CD, CMK, RDD, LM, and WM) read and approved the final manuscript.

## Pre-publication history

The pre-publication history for this paper can be accessed here:

http://www.biomedcentral.com/1471-244X/11/168/prepub
